# Systemic immune-inflammation index as a prognostic biomarker in critically ill dogs

**DOI:** 10.3389/fvets.2026.1900233

**Published:** 2026-07-28

**Authors:** Seong-Gyu Kim, Hyun-Jung Han

**Affiliations:** 1 Department of Veterinary Emergency and Critical Care Medicine, College of Veterinary Medicine, Konkuk University, Seoul, Republic of Korea; 2 KU Center for Animal Blood Medical Science, Konkuk University, Seoul, Republic of Korea

**Keywords:** critically ill dogs, intensive care unit, mortality, prognostic biomarker, systemic immune-inflammation index

## Abstract

Rapid and accurate prognostic assessment in critically ill dogs with systemic inflammation remains challenging. The Systemic Immune-Inflammation Index (SII), calculated as (neutrophil count × platelet count)/lymphocyte count, has been recognized as a biomarker for systemic inflammation and immune dysregulation in human medicine. However, its clinical relevance in veterinary emergency and critical care settings remains unclear. This study aimed to evaluate the clinical significance of the SII in critically ill dogs and establish its reference interval in healthy control dogs. This retrospective study analyzed 96 healthy control dogs and 61 critically ill dogs that presented to the intensive care unit (June 2023–January 2025). Complete blood count (CBC) parameters collected within 1 h of presentation were used to calculate the SII. CBC parameters and CBC-derived indices including SII were compared between critically ill dogs and healthy control dogs. Furthermore, CBC parameters, CBC-derived indices, and the Acute Patient Physiologic and Laboratory Evaluation (APPLE) score were compared between survivors and non-survivors. Receiver operating characteristic (ROC) curve analysis was performed to determine an optimal SII cutoff for predicting mortality and a 95% reference interval was established for healthy control dogs. Critically ill dogs had significantly higher median SII compared to healthy control dogs (1,997 vs. 516, *p* < 0.001). Non-survivors showed significantly elevated white blood cell count, neutrophil count, SII, neutrophil-to-lymphocyte ratio (NLR), and APPLEfast score compared to survivors. ROC curve analysis demonstrated that SII predicted mortality with moderate accuracy (area under the curve [AUC]: 0.806; cutoff: 2,628; sensitivity: 90%; specificity: 74.5%), with performance at least comparable to the APPLEfast score (AUC: 0.759; sensitivity: 90%; specificity: 64.7%) in this cohort. The 95% reference interval for SII in healthy control dogs was 180–1,392. In conclusion, SII was significantly increased in critically ill dogs, and values above the cutoff were associated with an increased mortality risk. These findings suggest that the SII is a valuable, novel indicator for prognostic evaluation in critically ill canine patients; however, further validation in larger cohorts is warranted.

## 1 Introduction

Rapid and accurate assessment of disease severity and prognosis represents one of the most critical clinical challenges in veterinary emergency and critical care medicine. To address this need, various clinical scoring systems have been implemented as essential tools for prompt evaluation. Although these systems are widely utilized, each possesses distinct advantages and inherent limitations. For instance, the Systemic Inflammatory Response Syndrome (SIRS) criteria offer simple application and high sensitivity but suffer from low specificity, raising concerns about overdiagnosis (1, 2). The Sequential Organ Failure Assessment (SOFA) and quick SOFA (qSOFA) scores, which are widely used in human medicine, have not yet been sufficiently validated in veterinary clinical settings (3, 4). The Acute Patient Physiologic and Laboratory Evaluation (APPLE) score is considered the most comprehensive and validated evaluation tool in veterinary medicine (5, 6). However, the calculation of the APPLE score requires 10 variables for the APPLEfull version or 5 variables for the APPLEfast version, making it difficult to implement immediately in emergency scenarios.

Prognosis in critically ill dogs is known to be closely related to complex interactions between systemic inflammatory responses and immune regulatory mechanisms (2, 7). Neutrophils, lymphocytes, and platelets play key roles in these pathophysiological processes, each with distinct immunological functions. Neutrophils are core cells of the innate immune system and play crucial roles in pathogen elimination and induction of inflammatory responses (8). In contrast, lymphocytes serve as the main component of the adaptive immune system. Although platelets are traditionally recognized for their role in hemostasis, they have recently been shown to actively participate in inflammation by interacting with immune cells and secreting inflammatory cytokines (9).

Based on these pathophysiological insights, inflammatory indices have been developed from routine complete blood count (CBC) results. Among the most widely studied are the neutrophil-to-lymphocyte ratio (NLR) and the platelet-to-lymphocyte ratio (PLR), which have shown prognostic value in both human and veterinary medicine (10–14). Recently, the Systemic Immune-Inflammation Index (SII) has been proposed in human medicine as an integrated inflammatory marker. It is calculated as (neutrophil count × platelet count)/lymphocyte count and is considered a comprehensive index that simultaneously reflects systemic inflammation and immunosuppression (15). SII has been actively studied as a prognostic biomarker across a broad range of diseases in human medicine, including cancer, cardiovascular diseases, and inflammatory disorders such as rheumatoid arthritis. It has demonstrated utility in predicting disease severity, treatment response, and long-term outcomes (16–20).

Despite its established value in human medicine, the application of the SII in veterinary emergency and critical care has only recently begun to be explored, and available evidence remains confined to individual disease entities rather than the general critically ill population. For example, CBC-derived inflammatory indices including the SII have been evaluated in cats with arterial thromboembolism (21) and in dogs with parvoviral enteritis complicated by systemic inflammatory response syndrome (22). To our knowledge, however, the SII has not yet been systematically investigated across a heterogeneous population of dogs admitted to an intensive care unit (ICU). This study aims to evaluate the clinical utility of the SII in dogs presented to the ICU. We hypothesized that SII would be significantly elevated in critically ill dogs compared to healthy controls and would effectively distinguish survivors from non-survivors. Furthermore, a reference interval for SII was calculated based on the control group to establish normal ranges in healthy individuals.

## 2 Materials and methods

### 2.1 Study design and animal selection

This study was designed as a retrospective observational study analyzing electronic medical records from Konkuk University Veterinary Medical Teaching Hospital during the study period (June 2023–January 2025). The study protocol was approved by the Institutional Animal Care and Use Committee (IACUC) of Konkuk University (Approval No. KU24251).

Healthy control dogs (*n* = 96) were selected from candidates presenting to the Canine Blood Donation Center between June and October 2024. To ensure a strictly healthy baseline, eligibility was determined based on the American Society for Veterinary Clinical Pathology (ASVCP) guidelines (23). Candidates were excluded if they met any of the following criteria: (1) a history of systemic illness, recent vaccination, or surgery; (2) clinically significant findings on physical examination, such as palpable masses, dermatologic lesions, or heart murmurs; and (3) laboratory abnormalities on a standardized screening panel comprising a complete blood count (CBC; ProCyte Dx, IDEXX Laboratories) and plasma biochemistry (Catalyst One, IDEXX Laboratories). Exclusionary findings were: a positive result for vector-borne pathogens (*Anaplasma* spp., *Borrelia* spp., *Ehrlichia* spp., *Dirofilaria immitis*, or *Babesia gibsoni*); anemia, defined as a hematocrit below the lower reference limit of the analyzer (<37.3%; reference interval 37.3–61.7%); or a clinically relevant elevation of hepatic enzymes, defined as alanine aminotransferase (reference interval 17–78 U/L) and/or alkaline phosphatase (reference interval 13–83 U/L) exceeding twice the upper reference limit in conjunction with a history compatible with hepatic involvement. The full selection and exclusion framework used to define this clinically healthy reference population is reproduced in Supplementary material Appendix 1.

Critically ill dogs were identified from patients admitted to the ICU between June 2023 and January 2025. Inclusion required the fulfillment of at least two Systemic Inflammatory Response Syndrome (SIRS) criteria—based on abnormalities in temperature, heart rate, respiratory rate, or white blood cell count— and the clinical judgment of a professor in the Department of Emergency and Critical Care (ECC) at a veterinary college, together with clinicians enrolled in an ECC residency program, each with more than 5 years of clinical experience. Patients were excluded if they: (1) had incomplete medical records; (2) were admitted solely for surgical intervention; (3) had received immunomodulatory agents (e.g., glucocorticoids, mycophenolate, or cyclosporine) or blood transfusions within the preceding month; or (4) were diagnosed with primary endocrine or hematologic disorders known to directly alter cell counts, such as hyperadrenocorticism or immune-mediated cytopenias. Ultimately, 61 dogs were included and categorized into survivors or non-survivors based on their clinical outcome. No restriction was applied to the underlying disease category so that the cohort would reflect the heterogeneous case mix typically encountered in a general ICU population. Because different disease processes may influence CBC parameters and the SII to differing degrees, the potential effect of this heterogeneity is addressed in the Discussion and Limitations.

### 2.2 Sample collection and laboratory analysis

Because this was a retrospective study, sampling times could not be standardized prospectively. Instead, for every case we cross-checked two timestamps recorded in the electronic medical record—the time of presentation and the time at which the blood sample was collected and analyzed—and included only cases in which blood was drawn and tested within 1 h of presentation. Blood for the complete blood count (CBC) and vector-borne pathogen screening (SNAP 4Dx) was collected into K2EDTA tubes (BD Microtainer® Tubes with K2E [K2EDTA]; Becton, Dickinson and Company, Franklin Lakes, NJ, USA), and blood for biochemical analysis was collected into lithium heparin tubes (BD Microtainer® Capillary Blood Collection Tube, Lithium Heparin; Becton, Dickinson and Company) for plasma biochemical analysis. All hematological and biochemical analyses were performed using the same automated systems (ProCyte Dx and Catalyst One, IDEXX Laboratories, Westbrook, ME, USA). To ensure the highest accuracy of the cellular indices, manual differential counts were performed by experienced clinical pathologists. These manual counts were mandated if the automated analyzer detected band neutrophils, dot-plot abnormalities, or thrombocytopenia. In these instances, manual count results were used for the final analysis. The APPLEfast scores were calculated as previously described (5). This score ranges from 0 to 50 points. On chart review, the corresponding mentation score was assigned when the patient’s mentation was described as follows: 0 for bright, alert, responsive (BAR) or no remarkable findings (NRF); 1 for quiet, alert, responsive (QAR); 2 for depressed or obtunded; 3 for stupor; and 4 for semi-coma or coma. The APPLEfast score comprises five variables—glucose, albumin, lactate, platelet count, and mentation score; the complete scoring scheme and the mentation score calculation applied in this study are provided in Supplementary material Appendix 2 [adapted from (5)].

Based on the absolute neutrophil, lymphocyte, and platelet counts, the following inflammatory indices were calculated:


$$\begin{array}{l} \text{Systemic Immune}-\text{Inflammation Index}\;(\mathrm{SII})\\ =\frac{\text{Neutrophil count}\times \text{Platelet count}}{\text{Lymphocyte count}} \end{array}$$

$$\text{Neutrophil}-\text{to}-\text{Lymphocyte ratio}\;(\mathrm{NLR})=\frac{\text{Neutrophil count}}{\text{Lymphocyte count}}$$

$$\text{Platelet}-\text{to}-\text{Lymphocyte ratio}\;(\mathrm{PLR})=\frac{\text{Platelet count}}{\text{Lymphocyte count}}$$

### 2.3 Statistical analysis

Statistical analyses were performed using SPSS Statistics 30.0 (IBM Corp., Armonk, NY, USA) and MedCalc® 23.2.0 (MedCalc Software Ltd., Ostend, Belgium). All statistical significance was set at *p* < 0.05. Normality was assessed using histograms and the Kolmogorov–Smirnov test (sample size ≥ 50) or Shapiro–Wilk test (sample size < 50). As most variables showed non-normal distributions, the Mann–Whitney *U* test was used for between-group comparisons. The Mann–Whitney *U* effect size (*r*) was calculated to quantitatively assess the magnitude of between-group differences and interpreted as small (0.1 ≤ *r* < 0.3), medium (0.3 ≤ *r* < 0.5), or large (*r* ≥ 0.5), according to Cohen (24). Receiver operating characteristic (ROC) curve analysis was performed to evaluate the predictive power of SII and other biomarkers for mortality. Optimal cutoff values were determined based on Youden’s index, and the area under the curve (AUC) was interpreted according to established criteria (25).

The 95% reference interval for SII was established following the ASVCP guidelines (23). Due to the non-normal distribution of SII in the 96 healthy controls, data were log-transformed. After confirming normality of the transformed data, the reference interval, together with the 90% confidence intervals (CIs) of its lower and upper limits, was calculated using the robust method with bootstrapping, and all values were back-transformed to the original scale for presentation. Following ASVCP recommendations, outliers identified by the Tukey method were included in the final analysis, as no clinical justification for exclusion was identified, ensuring the RI reflects the natural biological variation of the population.

## 3 Results

### 3.1 Population characteristics

The baseline clinical and demographic characteristics of the study population, along with the primary clinical diagnoses of the critically ill group, are summarized in Supplementary materials S1, S2, respectively. Individual-level data for all study animals, including complete blood count parameters and CBC-derived indices, are provided in Supplementary material S3 (healthy control dogs) and Supplementary material S4 (critically ill dogs). Most parameters exhibited non-normal distributions; therefore, all continuous variables are presented as median and range. The median age was significantly higher in the critically ill group compared with controls (8.0 years [range 0.3–19.0] vs. 4.0 years [range 1.0–8.0], *p* < 0.001). Body weight also differed markedly between the two groups: the healthy control group had a substantially higher median body weight (30.0 kg; range 17.0–51.0) than the critically ill group (7.0 kg; range 2.0–50.0) (*p* < 0.001). Reflecting the recruitment of blood donors, the control group was predominantly composed of large breeds, most notably Labrador Retrievers (*n* = 38) and Golden Retrievers (*n* = 20). In contrast, the critically ill group represented a more diverse distribution of 25 breeds, with a higher prevalence of small-breed dogs such as Miniature Poodles (*n* = 10), Chihuahuas (*n* = 4), and Pomeranians (*n* = 4). With respect to sex distribution, males (castrated and intact combined) predominated in the healthy control group (58/96, 60.4%), whereas the critically ill group showed a more balanced distribution (males 30/61, 49.2%; females 31/61, 50.8%). The proportion of neutered animals (castrated males and spayed females) was higher in the control group (85/96, 88.5%) than in the critically ill group (45/61, 73.8%). Primary diagnoses in the critically ill dogs were predominantly neoplastic (18.0%), infectious (14.8%), gastrointestinal (13.1%), and musculoskeletal (11.5%) diseases (Supplementary material S2). Among the 61 critically ill dogs, the overall survival rate to discharge was 83.6% (*n* = 51). The non-survivor group (*n* = 10, 16.4%) consisted of dogs that died naturally (*n* = 6) or underwent euthanasia (*n* = 4) during hospitalization.

### 3.2 Reference interval establishment of SII in healthy dogs

In the healthy control cohort, the raw SII values ranged from 198 to 1,995, with a median of 516 and a geometric mean of 524. The established 95% reference interval was 180–1,392, with 90% CIs of 158–209 and 1,180–1,636 for the lower and upper limits, respectively. Evaluation of the proportion of individuals exceeding the upper reference limit showed that only 6.25% (6/96) of healthy controls exceeded this threshold, whereas 59.0% (36/61) of critically ill dogs presented with values above it.

### 3.3 CBC parameters and CBC-derived indices

Significant differences in major hematological parameters were observed between the control and critically ill groups (Table 1).

**Table 1:** Comparison of complete blood count (CBC) parameters and CBC-derived indices between healthy control dogs and critically ill dogs.

Variable	Healthy control dogs (*n* = 96)	Critically ill dogs (*n* = 61)	Reference interval	*p*-value	Effect size (*r*)
WBC (×10^3^/μL)	8.51 (5.31–16.63)	13.24 (4.27–98.04)	5.05–16.76	<0.001	0.460
NEU (×10^3^/μL)	5.22 (3.14–13.79)	10.22 (3.59–67.65)	2.95–11.64	<0.001	0.554
LYM (×10^3^/μL)	2.17 (0.95–4.62)	1.54 (0.09–4.35)	1.05–5.10	<0.001	0.316
PLT (×10^3^/μL)	210 (113–490)	302 (17–904)	148–484	<0.001	0.449
SII	516 (198–1,995)	1,997 (136–26,216)	180–1,392^*^	<0.001	0.671
NLR	2.37 (1.10–8.57)	5.50 (1.40–49.00)	N/A	<0.001	0.641
PLR	96.12 (39.18–330.39)	195.11 (7.12–3,266.98)	N/A	<0.001	0.512

Values are presented as median (minimum–maximum).

Reference intervals were based on values provided by the IDEXX ProCyte Dx automated hematology analyzer.

^*^Reference interval for SII was established in the present study.

*p*-values were calculated using the Mann–Whitney *U* test. Results with *p*-values less than 0.05 were considered statistically significant.

LYM, lymphocyte count; NEU, neutrophil count; NLR, Neutrophil-to-Lymphocyte Ratio; PLR, Platelet-to-Lymphocyte Ratio; PLT, platelet count; SII, systemic immune-inflammation index; WBC, white blood cell count; N/A, not applicable.

Throughout the Results, values in square brackets [] denote the range (minimum–maximum). Compared to the control group, the critically ill group exhibited significantly higher median total white blood cell count (13.24 [4.27–98.04] vs. 8.51 [5.31–16.63] × 103/μL; *p* < 0.001; effect size *r* = 0.460), neutrophil count (10.22 [3.59–67.65] vs. 5.22 [3.14–13.79] × 103/μL; *p* < 0.001, *r* = 0.554), and platelet counts (302 [17–904] vs. 210 [113–490] × 103/μL; *p* < 0.001; *r* = 0.449). In contrast, lymphocyte count was significantly lower in the critically ill group (1.54 [0.09–4.35] vs. 2.17 [0.95–4.62] × 103/μL; *p* < 0.001; *r* = 0.316).

**Figure 1:**
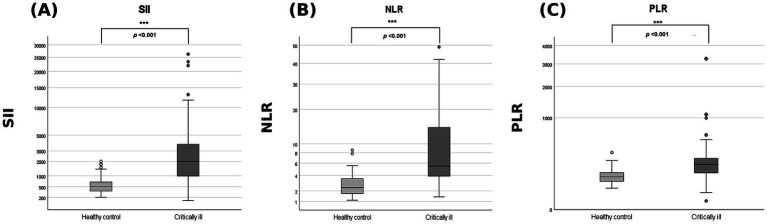
Boxplot comparison of **(A)** systemic immune-inflammation index (SII), **(B)** neutrophil-to-lymphocyte ratio (NLR), and **(C)** platelet-to-lymphocyte ratio (PLR) values between healthy control dogs (*n* = 96) and critically ill dogs (*n* = 61). The horizontal line within each box indicates the median value. Mild outliers (values > 1.5 × interquartile range [IQR] from the quartiles) are shown as open circles (○), and extreme outliers (values > 3 × IQR) are marked with diamonds (◆). The *y*-axis is presented on a logarithmic scale to account for skewed distributions. Asterisks above the brackets denote statistically significant differences between groups (^***^p < 0.001, Mann–Whitney U test).

CBC-derived indices showed even more pronounced differences. SII was significantly higher in critically ill dogs compared to controls (1,997 [136–26,216] vs. 516 [198–1,995]; *p* < 0.001). Notably, SII demonstrated the largest effect size among all measured parameters (*r* = 0.671), indicating a greater discriminative ability between groups. NLR was also significantly elevated in the critically ill group (5.50 [1.40–49.00] vs. 2.37 [1.10–8.57]; *p* < 0.001; *r* = 0.641), as was PLR (195.11 [7.12–3,266.98] vs. 96.12 [39.18–330.39]; *p* < 0.001; *r* = 0.512). The distributions of SII, NLR, and PLR values in healthy control dogs and critically ill dogs are illustrated in Figure 1, demonstrating a clear separation between groups, with SII showing the most pronounced difference.

### 3.4 Comparison between survivors and non-survivors

Comparison between survivors (*n* = 51) and non-survivors (*n* = 10) revealed that non-survivors had significantly higher median total white blood cell count (23.40 [4.27–65.50] vs. 12.00 [5.32–98.04] × 103/μL; *p* = 0.005) and neutrophil counts (20.72 [3.59–59.61] vs. 8.80 [3.77–67.65] × 103/μL; *p* = 0.004) (Table 2). However, no statistically significant differences were observed in lymphocyte (*p* = 0.785) or platelet counts (*p* = 0.572).

**Table 2:** Comparison of population characteristics, CBC parameters, CBC-derived indices, and APPLEfast score between survivors and non-survivors among critically ill dogs.

Variable	Survivors (*n* = 51)	Non-survivors (*n* = 10)	Reference interval	*p*-value	Effect size (*r*)
Age (years)	8.0 (0.3–19.0)	10.0 (7.0–15.0)	N/A	0.145	0.187
Weight (kg)	7.0 (2.2–50.0)	5.9 (2.0–38.1)	N/A	0.489	0.089
WBC (×10^3^/μL)	12.00 (5.32–98.04)	23.40 (4.27–65.50)	5.05–16.76	0.005	0.359
NEU (×10^3^/μL)	8.80 (3.77–67.65)	20.72 (3.59–59.61)	2.95–11.64	0.004	0.369
LYM (×10^3^/μL)	1.52 (0.45–4.35)	2.08 (0.09–3.49)	1.05–5.10	0.785	0.035
PLT (×10^3^/μL)	302 (17–636)	310 (93–904)	148–484	0.572	0.072
SII	1,641 (136–23,324)	4,247 (1,092–26,216)	180–1,392^*^	0.002	0.389
NLR	4.92 (1.40–49.00)	17.35 (5.17–42.00)	N/A	0.003	0.377
PLR	195.11 (7.12–1,083.89)	175.81 (70.25–3,266.98)	N/A	0.770	0.037
APPLEfast Score	19 (10–35)	22.5 (17–29)	N/A	0.010	0.330

Values are expressed as median (minimum–maximum).

Reference intervals were based on values provided by the IDEXX ProCyte dx automated hematology analyzer.

^*^Reference interval for SII was established in the present study.

*p*-values were calculated using the Mann–Whitney *U* test. Results with *p*-values less than 0.05 were considered statistically significant.

APPLEfast, Acute Patient Physiologic and Laboratory Evaluation – FAST score; LYM, lymphocyte count; NEU, neutrophil count; NLR, Neutrophil-to-Lymphocyte Ratio; PLR, Platelet-to-Lymphocyte Ratio; PLT, platelet count; SII, Systemic Immune-Inflammation Index; WBC, white blood cell count; N/A, not applicable.

**Figure 2:**
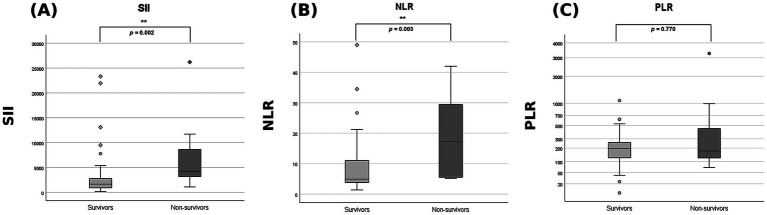
Boxplot comparison of **(A)** systemic immune-inflammation index (SII), **(B)** neutrophil-to-lymphocyte ratio (NLR), and **(C)** platelet-to-lymphocyte ratio (PLR) between survivors (*n* = 51) and non-survivors (*n* = 10) among critically ill dogs. The horizontal line within each box represents the median value. Mild outliers (values > 1.5 × interquartile range [IQR] from the quartiles) are shown as open circles (○), and extreme outliers (values > 3 × IQR) are marked with diamonds (◆). The *y*-axis of the PLR graph is displayed on a logarithmic scale to accommodate skewed distributions. Asterisks above the brackets denote statistically significant differences between groups (^**^p < 0.01, Mann–Whitney *U* test).

Regarding CBC-derived indices, the median SII in non-survivors was approximately 2.6-fold higher than in survivors (4,247 [1,092–26,216] vs. 1,641 [136–23,324]; *p* = 0.002), demonstrating the largest effect size (*r* = 0.389) among all variables compared. NLR was also significantly higher in non-survivors (17.35 [5.17–42.00] vs. 4.92 [1.40–49.00]; *p* = 0.003), whereas PLR did not differ significantly between groups (*p* = 0.770). The distribution of SII, NLR, and PLR values between survivors and non-survivors among critically ill dogs is depicted in Figure 2. Additionally, non-survivors exhibited significantly higher APPLEfast scores (22.5 [17–29] vs. 19 [10–35]; *p* = 0.010), although the statistical significance was less pronounced than that of the SII.

### 3.5 ROC curve analysis and diagnostic accuracy

**Figure 3:**
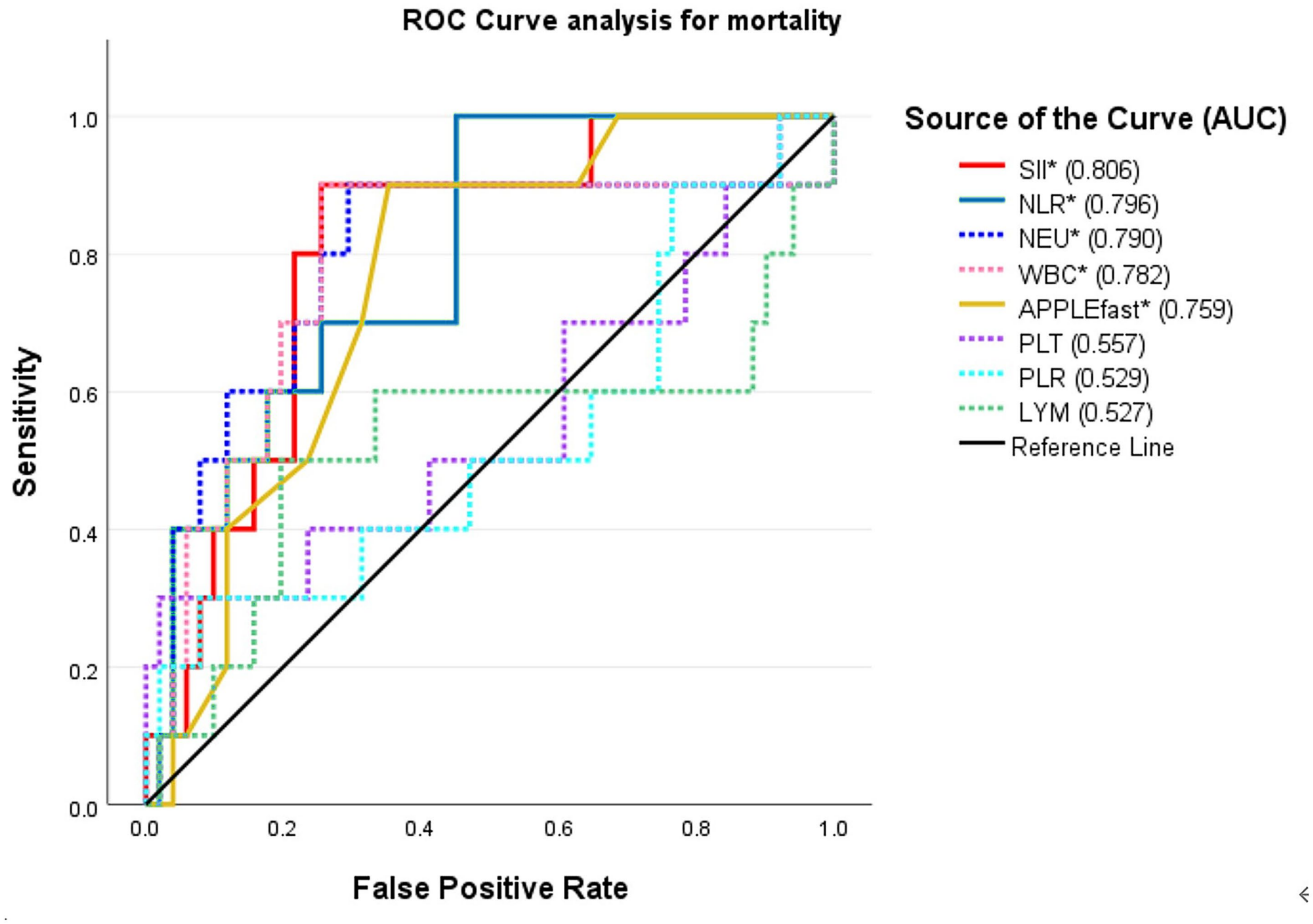
Receiver operating characteristic (ROC) curve analysis for mortality prediction in critically ill dogs. The curves represent the diagnostic performance of each variable: APPLEfast score, lymphocyte count (LYM), neutrophil count (NEU), neutrophil-to-lymphocyte ratio (NLR), platelet-to-lymphocyte ratio (PLR), platelet count (PLT), systemic immune-inflammation index (SII), and white blood cell count (WBC). Values in parentheses next to each variable in the legend indicate the area under the curve (AUC). Asterisks (*) denote variables that were statistically significant predictors of mortality (*p* < 0.05). The diagonal black line indicates the reference line (no discrimination).

ROC curve analysis identified total white blood cell count, neutrophil count, SII, NLR, and APPLEfast score as significant predictors of mortality (*p* < 0.05) (Figure 3). At their optimal cutoffs, total white blood cell count (17.87 × 10^3^/μL) yielded a sensitivity of 90% and a specificity of 74.5% with an AUC of 0.782 (95% CI: 0.601–0.964; *p* = 0.002); neutrophil count (14.19 × 10^3^/μL), 90 and 70.6% with an AUC of 0.790 (95% CI: 0.607–0.974; *p* = 0.002); NLR (5.15), 100 and 54.9% with an AUC of 0.796 (95% CI: 0.664–0.928; *p* < 0.001); and APPLEfast (score of 20), 90 and 64.7% with an AUC of 0.759 (95% CI: 0.622–0.896; *p* < 0.001). In contrast, lymphocyte count (AUC 0.527, *p* = 0.826), platelet count (AUC 0.557, *p* = 0.617), and PLR (AUC 0.529, *p* = 0.790) did not demonstrate significant predictive ability. Among these, SII demonstrated the highest AUC of 0.806 (95% CI: 0.675–0.937), indicating moderate accuracy. The optimal cutoff value for SII was determined to be 2,628, yielding a sensitivity of 90% and a specificity of 74.5%. Notably, the SII appeared to provide better prognostic performance than the APPLEfast score (AUC = 0.759) in this cohort, characterized by a higher AUC and markedly better specificity (74.5% vs. 64.7%) while achieving identical sensitivity (90%). Furthermore, while the NLR exhibited maximum sensitivity (100%), its specificity (54.9%) was markedly lower than that of SII.

## 4 Discussion

This study evaluated the clinical utility of the Systemic Immune-Inflammation Index (SII) as a prognostic biomarker in critically ill dogs. Our findings demonstrate that SII is a practical and effective tool for prognostic assessment in veterinary emergency and critical care medicine. Consistent with our hypothesis, critically ill dogs exhibited a nearly fourfold increase in median SII values compared to healthy controls (1,997 vs. 516; *p* < 0.001). Notably, the SII demonstrated the largest effect size (*r* = 0.671) among all analyzed variables, suggesting a potentially greater discriminative ability compared to individual hematological parameters. Furthermore, SII significantly distinguished non-survivors from survivors, achieving a moderate-to-high prognostic accuracy with an AUC of 0.806. This performance suggests that the SII, as an integrated biomarker, effectively captures the multidimensional nature of systemic dysregulation in critical illness.

The SII reference interval established in this study (180–1,392) provides a critical baseline for veterinary clinical interpretation. By establishing this normative range first, we provide a standardized framework to quantify the degree of immune-inflammatory dysregulation in diseased states. The stark disparity in the proportion of individuals exceeding the upper limit—only 6.25% of healthy dogs versus 59% of critically ill dogs—underscores the high clinical sensitivity of this index in identifying systemic inflammation. While the age and breed differences between our control and critically ill groups must be considered, the magnitude of SII elevation in the patient group far exceeded reported biological variations. Notably, the control group had a median age of 4 years, whereas the critically ill cohort was older (median: 8 years). Regarding the potential impact of age on hematological parameters, previous studies have yielded conflicting results. For instance, Lee and colleagues reported no statistically significant differences in neutrophil, lymphocyte, and platelet counts between young (1–3 years) and old (7–10 years) dogs (26). Conversely, Strasser and colleagues observed that old dogs (6–13.5 years) exhibited significantly lower neutrophil and lymphocyte counts, whereas platelet counts were significantly higher compared to young dogs (0.5–5 years) (27). Given this inconsistency in the literature, a definitive conclusion regarding the influence of age on SII cannot be drawn from the present cohort. Furthermore, although certain sighthounds are known to have lower baseline white blood cell, neutrophil, and platelet counts, these breeds were not represented in this study, thereby minimizing potential confounding from this specific breed-related variation (28). In addition to age and breed, the two groups differed substantially in body weight (control median 30.0 kg vs. critically ill 7.0 kg), reflecting the predominance of large-breed blood donors in the control group and small-breed dogs in the ICU cohort. Although we are not aware of consistent evidence that body size per se alters the SII, breed- and size-associated differences in baseline leukocyte and platelet counts cannot be fully excluded as a source of residual confounding. These demographic disparities are acknowledged as a limitation of the present study.

Consequently, despite the demographic differences, the magnitude of SII elevation in the patient group clearly exceeded normal biological variability, suggesting that values exceeding 1,392 can be reliably interpreted as indicative of significant systemic immune-inflammatory dysregulation, transcending age or breed-related shifts.

The pronounced elevation of the SII in critically ill dogs reflects the complex interplay between systemic inflammatory responses and immune regulatory mechanisms. The neutrophilia observed in this study, driven by bone marrow mobilization and delayed apoptosis during SIRS or sepsis, serves as a primary effector of the innate immune response (8, 10). Concurrently, lymphopenia—likely due to glucocorticoid-mediated redistribution and apoptotic depletion—acts as a hallmark of immune suppression (29, 30). In critical conditions such as sepsis or trauma, CD4^+^ and CD8^+^ T cells rapidly decline due to apoptosis, while regulatory T cells become predominant and exert immunosuppressive effects (30). Furthermore, reactive thrombocytosis, often induced by pro-inflammatory cytokines like interleukin-6 (31, 32), amplifies systemic inflammation by promoting neutrophil extracellular trap formation and modulating cytokine release (33). By integrating these three distinct cellular lineages, the SII serves as a multi-dimensional biomarker that effectively captures the shift from a hyper-inflammatory state to subsequent immunologic exhaustion more comprehensively than individual cell counts alone.

A key finding of this study is the SII’s ability to discriminate between survivors and non-survivors among critically ill patients. The median SII in non-survivors was approximately 2.6-fold higher than in survivors (4,247 vs. 1,641; *p* = 0.002), yielding the largest effect size (*r* = 0.389) among all parameters. Comparison of CBC parameters between survivors and non-survivors revealed that the elevation in SII was primarily driven by neutrophilia. Non-survivors exhibited significantly higher neutrophil counts compared to survivors, indicative of sustained systemic inflammation. Prolonged neutrophil activation drives vascular unresponsiveness and compromises endothelial integrity, ultimately leading to multiple organ failure and death (2). Furthermore, in advanced stages of SIRS or sepsis, neutrophil function becomes compromised with diminished bacterial clearance and attenuated reactive oxygen species production, thereby increasing vulnerability to secondary infections (34).

Interestingly, while individual lymphocyte and platelet counts did not differ significantly between survivors and non-survivors, their mathematical integration into the SII magnified subtle imbalances, resulting in a robust prognostic signal. In the present study, significant lymphopenia and reactive thrombocytosis were hallmarks that distinguished critically ill dogs from healthy controls. However, the absence of such statistical differences between survivors and non-survivors suggests that once a threshold of critical illness is reached, individual cell counts may reach a “plateau” or exhibit high inter-individual variability that obscures their direct prognostic utility (35, 36). The SII overcomes this limitation by compounding these individual shifts; even if the decrease in lymphocytes or the increase in platelets is not statistically significant on its own within the ICU cohort, their simultaneous presence in the numerator and denominator of the SII formula geometrically amplifies the resulting value. Ultimately, the marked neutrophilia in non-survivors acts as the decisive factor in this equation. As the neutrophil count is a direct multiplier in the numerator, its substantial elevation—indicative of sustained systemic inflammation (2)—elevates the SII beyond the risk threshold. Thus, the SII functions as a sensitive “stress-integrator” that reflects the cumulative burden of dysregulation, potentially offering added prognostic value where individual cellular markers fall short in high-stakes emergency settings.

This study has several limitations inherent in its retrospective, single-center design. First, the control and critically ill groups differed significantly in age, body weight, and breed distribution, which may confound hematological comparisons despite the large magnitude of SII elevation observed. Second, the critically ill cohort comprised a highly heterogeneous mix of diseases (e.g., neoplastic, infectious, gastrointestinal, and musculoskeletal), and different disease processes may affect the SII to differing degrees; the present design cannot disentangle these disease-specific effects. Third, the number of non-survivors was small (*n* = 10), which limited statistical power and precluded multivariable analysis to adjust for potential confounders; the reported cutoff should therefore be regarded as preliminary. Fourth, 4 of the 10 non-survivors were euthanized, and because the decision to euthanize may be influenced by factors other than the underlying physiologic severity, this may have affected the mortality endpoint. Fifth, only a single SII value obtained at presentation was analyzed; serial SII measurements during hospitalization, which could capture dynamic changes and treatment response, were not available. Finally, the exclusion of dogs receiving immunomodulatory drugs—while necessary to prevent confounding—limits the applicability of these findings to patients already on such treatments. Despite these constraints, to our knowledge this is the first study to systematically evaluate the SII across a general ICU population in veterinary emergency and critical care medicine, and it provides a practical, accessible prognostic biomarker that warrants prospective validation.

Future research should prioritize prospective studies involving larger, multi-center cohorts to establish external validity and refine breed-specific considerations. Furthermore, serial evaluation of the SII during hospitalization may offer valuable insights into treatment responsiveness and dynamic patient progression, which was beyond the scope of this cross-sectional analysis.

The SII is a simple, CBC-derived index that offers immediate availability for clinical decision-making in veterinary critical care. Based on our findings, we recommend implementing the identified SII cutoff of 2,628 as a risk stratification threshold for intensive monitoring and aggressive intervention in critically ill dogs. The established reference interval (180–1,392) provides a robust foundation for clinical interpretation, though clinicians should always integrate the SII within the broader clinical context, considering medication history and concurrent hematologic disorders. In conclusion, the SII may provide a cost-effective alternative or adjunct to existing scoring systems, facilitating better resource allocation and improved prognostic assessment in high-stakes clinical settings.

## Data Availability

The original contributions presented in the study are included in the article/Supplementary material, further inquiries can be directed to the corresponding author.
