# Supplementary material for: Systemic immune-inflammation index as a prognostic biomarker in critically ill dogs
Source: Front Vet Sci. 2026 Jul 28;13:1900233. doi: 10.3389/fvets.2026.1900233 (PMC13457013; doi:10.3389/fvets.2026.1900233)
Supplement: Supplementary file 1 [file Data_Sheet_1.PDF]

Supplementary Material_Appendices

Supplementary Material_Appendix 1. Criteria used to define the clinically healthy control (reference) population

Selection and exclusion of the healthy control dogs were guided by the American Society for Veterinary Clinical Pathology (ASVCP) reference interval guidelines (Friedrichs et al., 2012; doi: 10.1111/vcp.12006). The table below reproduces the ASVCP framework of selection and exclusion criteria for reference individuals (ASVCP Guidelines, Table 2), which was used as the conceptual basis for constructing our healthy control group and for establishing the SII reference interval reported in this study.

Classification	Category	Example
Selection criteria*		
Biological	Age	Neonate, juvenile, adult
	Sex	Female, male, altered
	Breed	Holstein, Angus
	Strain	Cynomolgus monkey from Mauritius or Southeast Asia
	Supplier	Sprague-Dawley rats from 2 different suppliers
Clinical	History	No signs of illness in the 2 weeks preceding or following sample collection
	Preventative care	Vaccination, routine anthelmintics
	Health	Physical examination
	Diagnostic evaluation	Routine hematology, biochemistry, urinalysis, imaging studies
	Husbandry	Farmed, free-living, diet
Geographic	Location	Coastal, temperate, mountain, specific state or region
	Environment	Ambient temperature
Exclusion criteria		
Biological	Metabolic	Fasted or non-fasted, intense exercise, high stress
	Cell damage	Traumatic venipuncture, physical or chemical restraint
Physiologic	—	Illness, medications, lactation*, pregnancy*
Medications	—	Hormones or growth promoters, enzyme inducers (corticosteroids or antileptics)

*Reproduced from Friedrichs KR, et al. ASVCP reference interval guidelines: determination of de novo reference intervals in veterinary species and other related topics. Vet Clin Pathol. 2012;41(4):441–453 (Table 2). *May be used as partitioning criteria.*

Supplementary Material_Appendix 2. APPLEfast score and mentation score calculation

The APPLEfast (Acute Patient Physiologic and Laboratory Evaluation – FAST) score comprises five variables: glucose, albumin, lactate, platelet count, and mentation score. For each variable, the points shown for the band containing the patient's value are assigned; a value of zero is ascribed for the central (reference) band, shaded below. The five values are summed for a total score (maximum 50).

Parameter	Band 1 (pts)	Band 2 (pts)	Band 3 (pts)	Band 4 (pts)	Band 5 (pts)
**Glucose (mmol/L)**	<4.6 (7)	4.6–5.6 (8)	5.7–9.0 (9)	9.1–15.0 (10)	>15.0 (0)
**Albumin (g/L)**	<26 (8)	26–30 (7)	31–32 (6)	33–35 (0)	>35 (2)
**Lactate (mmol/L)**	<2 (0)	2–8 (4)	8–10 (8)	>10 (12)	
**Platelet count (×10³/µL)**	<151 (5)	151–200 (6)	201–260 (3)	261–420 (0)	>420 (1)
**Mentation score**	0 (0)	1 (4)	2 (6)	3 (7)	4 (14)

Table adapted from Hayes G, et al. The Acute Patient Physiologic and Laboratory Evaluation (APPLE) score: a severity of illness stratification system for hospitalized dogs. J Vet Intern Med. 2010;24:1034–1047 (Figure A2, SI units).

Mentation score (Hayes et al., 2010, Table 3):

Score	Mentation (assessed at admission, before sedation/analgesic administration)
0	Normal
1	Able to stand unassisted, responsive but dull
2	Can stand only when assisted, responsive but dull
3	Unable to stand, responsive
4	Unable to stand, unresponsive
